# Interactions between areas of the cortical grasping network

**DOI:** 10.1016/j.conb.2011.05.021

**Published:** 2011-08

**Authors:** Marco Davare, Alexander Kraskov, John C Rothwell, Roger N Lemon

**Affiliations:** Sobell Department of Motor Neuroscience and Movement Disorders, UCL Institute of Neurology, Queen Square, WC1N 3BG, London, UK

## Abstract

Skilled grasp is a sensorimotor process requiring the brain to extract sensory cues from the environment to shape a motor command. Although a large body of literature has focused on which brain areas either integrate the visual object's properties or control the motor output, it is still unclear how grasp-related information is transferred from one area to another. Understanding interactions between brain areas is crucial for the study of visuomotor transformations. Recently, new advances in both human and non-human primates have shown it is possible to study cortico-cortical interactions during different task contexts. This sheds new light on how brain areas are integrated in a dynamic network for controlling grasping actions.

## Highlights

► The anterior intraparietal area (AIP) is crucial for the processing of grasp-related object properties. ► AIP receives visual information about graspable objects from both the dorsal and ventral stream. ► Reciprocal interactions between the ventral premotor (PMv) and primary motor cortex (M1) allow the motor command to be grasp-specific. ► AIP plays a causal role in influencing interactions between PMv and M1.

## Introduction

The hand is the principal organ through which we interact with our environment. Skilled hand function contributes to many different aspects of our daily life and is crucial for our technology, communication, culture and social interaction. The loss of hand function is devastating. In a survey of quadriplegic patients, the regaining of arm and hand function was ranked as most important [1].

Reaching out and grasping an object requires the processing of its precise location with respect to our hand and the integration of the object's intrinsic properties such as its size and shape. These visual attributes have then to be transformed into an appropriate motor command that will guide and shape our hand for efficient grasp of the object. The safe and efficient application of fingertip forces to the object requires that this command takes into account the biomechanical interactions all along the multi-articulate bony chain linking the proximal arm to the wrist, hand and phalanges.

Recent studies provide new insights about which brain areas are involved in the fast processing mechanisms underlying grasp, and how grasp-related visual and motor information is transferred between the involved areas. Moreover, recent findings suggest interactions between the object recognition system in the ‘ventral’ occipito-temporal stream and the system controlling goal-directed actions in the ‘dorsal’ occipito-parietal stream. These interactions could be important as a means of mediating the details of an object's properties to the dorsal stream in order to fine-tune the motor command for grasping it.

## What is represented in the different components of the visuomotor cortical network for skilled grasp?

The classical model of the neural control of reaching and grasping movements proposes that areas located in the antero-lateral portion of the intraparietal sulcus (IPS) integrate grasp-related information about an object whereas a more postero-medial region of the IPS contributes to the planning of reaching movements towards the object. The anterior intraparietal area (AIP) contains visual and visuomotor neurons that are activated by a particular type of grasp [2,3], while the medial intraparietal area (MIP) and V6A contain neurons associated with a particular direction of reach [4]. On the convexity of the inferior parietal lobule, areas PF, PFG and PG are also organised in a somatotopic gradient and show object-related sensorimotor properties related to mouth, hand and arm movements, respectively [5].

**Figure 1:**
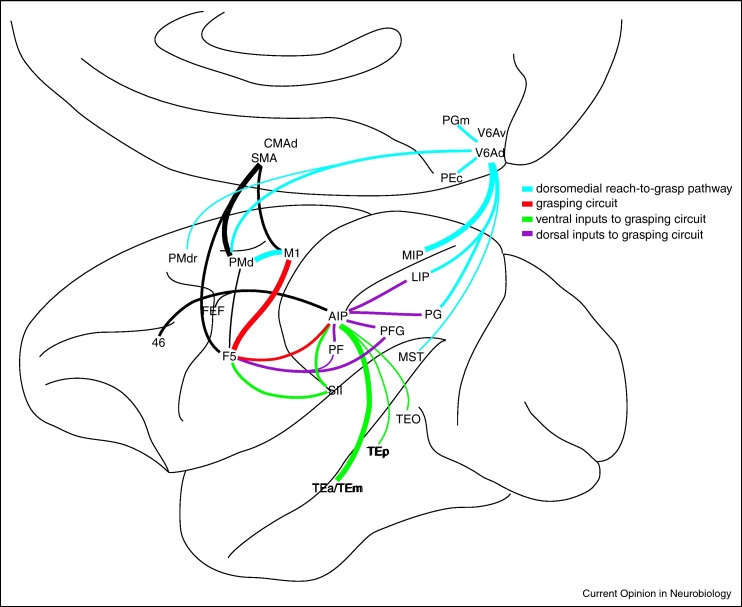
**Anatomical connections of the cortical grasping network based on tract tracing in non-human primates (from Grafton, 2010)**. Anatomic labelling is approximate. The anterior intraparietal area (AIP) is a key node for processing grasp-related object properties. AIP is part of the dorsolateral ‘grasping’ circuit (in red). It receives inputs from areas located in the dorsal stream (inferior parietal lobule [PF, PFG, PG] and the lateral intraparietal area [LIP], in purple) and from areas in the ventral stream (secondary somatosensory cortex [SII], infero-temporal [TEa/TEm, TEp, TEo] and medio-superior temporal lobule [MST], in green). These inputs provide AIP with real-time details about an object's properties together with stored knowledge about its identity. AIP makes reciprocal connections with ventral premotor area (PMv/F5) that in turn is reciprocally connected to the primary motor cortex (M1) hand area. These AIP–F5–M1 interactions are grasp-specific and crucial for controlling visually guided grasp. The dorsomedial ‘reach-to-grasp’ circuit (in blue) involves area V6A (see Ref. [14^•^]). It is connected with the medial intraparietal area (MIP), LIP, PG, MST, mesial parietal areas (PEc and PGm) and the dorsal premotor cortex (PMd, PMdr).

All these structures feature in modern views of the reach and grasp network (Figure 1; [6^••^]). In order to show grasp-related selectivity of neurons in many component areas of the network it has been necessary to test a wide range of grasps [2,7,8]. This approach first demonstrated that area F5, the rostral part of the ventral premotor cortex (PMv) in the macaque monkey, contains visuomotor and motor neurons that are selectively active while the animal is fixating and grasping objects of a particular shape using a particular range of grasps [8–10]. This kind of detailed study led to the concept of ‘canonical’ neurons in area F5 that are thought to form a motor repertoire of possible grasping actions [11]. In terms of the F5 signals that might be used to control a grasping prosthesis via a Brain–Machine Interface, it is important to note that not only spike activity but also local field potentials recorded from F5, which represent net excitatory and inhibitory dendritic synaptic potentials, were found to be grasp-specific during steady hold of an object [12^••^].

Some recent findings challenge the view that the reach and grasp components are processed independently. Fattori *et al.* (2010) have recently reported neurons in V6A whose activity is modulated by grasp type, and where the influence of visual inputs, reach activity and wrist orientation could be excluded [13,14^•^]. Thus although the classical view suggests that neurons in this area encode the direction of the arm towards different spatial locations, these recent findings suggest that area V6A may be involved in controlling both the reach and the grasp (blue in Figure 1). There is also evidence of grasp-specific activity from recordings in the dorsal premotor cortex (PMd/F2) [15,16], even though this is traditionally part of the ‘reaching’ circuit (dorsomedial pathway, in blue in Figure 1). Moreover, a grasp-specific representation within PMd is predicted by its neuroanatomical connectivity, with heavy interactions with digit representations in both PMv (F5) and M1 [17,18].

Of course biomechanical constraints mean that the execution of a particular type of grasp will be influenced by the position and orientation of the object in the workspace, and it is of interest to know whether ‘grasp-related’ activity in classically grasp-dominated areas is in fact influenced by object orientation. In recent papers it was shown that wrist orientation can strongly influence grasp-related activity in both AIP [19] and F5 [20]. Moreover, using transcranial magnetic stimulation (TMS) in humans, it has been found that the corticospinal excitability of particular hand muscles is modulated by the shoulder position; suggesting a flexible cortical drive to hand muscles depending on arm position [21]. In a task where the grip force has to be kept constant, the drive to both intrinsic and extrinsic hand muscles is modulated by wrist orientation [22]. Therefore, even if only the force generated by extrinsic hand muscles is influenced by different wrist angles, it seems that the motor system controls both intrinsic and extrinsic hand muscles as a synergistic group. Extending this notion raises the idea that commands related to the transport component, and involving proximal muscles, also influence the activity of distal muscles required for the grasp.

In humans, functional imaging studies also show evidence that the control of the reach and grasp components might not be independent. It has been found that areas in the dorsomedial pathway (V6A and PMd) were strongly coupled during grasping, in a similar fashion to the coupling of AIP and PMv in the dorsolateral circuit [23]. Interestingly, AIP and PMv were more coupled during grasp of a small object [23]. In addition, the AIP–PMv circuit showed strong coupling with the lateral occipital complex (LOC) in conditions where perceptual information about an object was crucial to achieve an appropriate grasp [24]. This suggests that the AIP–PMv circuit could incorporate physical details originating from the ventral visual stream to fine-tune the grasp.

This is a dynamic network, and plastic changes can occur when particular types of skilled grasp, such as the use of a tool, are learned for the first time [25^••^,26]. During tool-use learning in young macaques, Quallo *et al.* (2009) found learning-induced gray matter changes in the superior temporal sulcus, the second somatosensory area (SII) and the IPS, possibly reflecting strengthened interactions between the ventral and the dorsal visual streams (Figure 1).

TMS studies aiming at inducing virtual lesions in AIP showed a causal relationship between the AIP normal working and its role in grasp behaviour. Hand shaping and grip force scaling were affected following disruption of AIP by rTMS [27,28]. Moreover, rTMS of AIP also disrupts online adjustments of the grasp that are goal-dependent [29,30]. TMS disruption of human PMv leads to deficits in planning an accurate hand configuration and in predictive force scaling [28,31^•^].

We can conclude that the control of grasp relies on both the dorsomedial (blue in Figure 1) and dorsolateral (red in Figure 1) pathways. However, AIP seems to have a particular functional specialisation for grasp that is dependent on on-line visuomotor control. PMd may be more concerned in coupling the grasp to other aspects of the movement, such as reaching for the object [32] or lifting it after it has been grasped [31^•^]. Such a role could be supported by the presence within PMd of both distal and proximal arm muscles [17,18].

## Transfer of grasp-related information between areas of the grasping network

It is important to recognise that the characteristic properties of a given component of the cortical network are not intrinsic to that area but arise from its specific interactions with other members of the network. Because of their particular role in controlling grasping movements, the interactions between AIP, PMv and M1 have been the subject of recent intensive research. AIP and PMv are reciprocally connected and receive inputs from the ventral visual stream areas, including the lower bank of the superior temporal sulcus in the region of areas TEa/TEm and the middle temporal gyrus [33], and SII. Rapid access by the AIP–PMv circuit to object identity information stored in the ventral stream could be crucial to fine-tune the grasp so that it is appropriate for a particular object.

To explore how information about an object to be grasped is transferred within the human AIP–PMv–M1 circuit, two new techniques of paired-pulse and repetitive TMS have been developed. Using the paired-pulse approach it was found that PMv exerts grasp-specific facilitation of M1 [34^••^,35], in keeping with that first shown in the monkey [36,37]. Moreover, PMv–M1 interactions are driven by information about object properties provided by AIP [38^••^]. These results are important because they established that there is a causal transfer of information about object properties between AIP and PMv. Owing to the reciprocal nature of AIP–PMv connections [11,33], it is possible that ‘canonical’ neurons in PMv acquire their grasp-selective properties through rapid recurrent feedback loops between PMv and AIP. Moreover, if the motor command has to be updated, these recurrent loops would allow AIP to inform the motor output online, depending on the object's new properties. Indeed, Buch *et al.* (2010) found that the right PMv–left M1 interactions could mediate information about how to adjust the grasp as soon as 75 ms after the object changed [39].

Another paired-pulse TMS study, carried out in resting volunteers, found that the intensity of the conditioning stimulus modified its effect on the corticospinal excitability tested from M1 [40]. The recruitment of different PMv–M1 pathways by different TMS intensities might be influenced by the grasp context, bringing into play different neural populations involved in the task. Because the grasp-related information is represented in the PMv canonical neurons, a model of connectivity between PMv and M1 has been proposed in which the canonical neurons define a particular motor prototype by controlling the balance of inhibition/facilitation to complex muscle representations in M1 [38^••^]. These representations are now known to be complex and overlapping in nature, with multiple representation of a given muscle that probably underpins the huge repertoire of human grasping actions [41,42^••^,43].

Using TMS, it is also possible to investigate the time course of a particular cortico-cortical interaction during movement planning. Koch *et al.* (2010) tested interactions between anterior and caudal regions of the IPS with M1 when grasping objects in central or peripheral space. They found that the caudal part of IPS interacted with M1 early during the preparation of movements requiring a whole hand grasp in the peripheral space. By contrast, the anterior portion of IPS interacted with M1 at a later stage and only for a precision grip, irrespective of object location [44]. The pathways mediating these interactions are not obvious, since there are no known direct projections from the caudal IPS to M1.

Interactions between natural activity in premotor and motor cortex have been detected in monkey studies. Stark *et al.* (2008) showed that correlations between small populations of neurons measured by multiunit activity recorded in different parts of premotor cortex carry information about combination of reach and grasp [45^•^]. Kraskov *et al.* (2010) demonstrated that interactions between LFPs and single units recorded in area F5 and M1 changed during a grasping task. They also found an asymmetric relationship between LFPs in one area and single unit activity in another area, that is LFPs in M1 are much more coherent with single unit activity in F5 than LFPs in F5 with single units in M1 [46]. This asymmetry might be speculated to reflect the transfer of information from F5 to M1 related to the selection of the appropriate grasp.

Because the corticospinal projections from PMv to the lower cervical segments innervating hand muscles are scarce [17,47], it has been hypothesised that PMv controls grasp indirectly via M1. Thus PMv contributes to the control of hand shape through its corticocortical connections with M1 [36]. The facilitation by F5 of descending corticospinal volleys from M1 is abolished by reversible inactivation of M1 [36,48,49^••^]. It is well known that intracortical stimulation of F5 evokes characteristic digit movements; these movements are also abolished by reversible inactivation of M1 [49^••^].

## Conclusions

This brief review has highlighted the complexity of the cortical grasping network, and this undoubtedly reflects the biomechanical complexity of the reach-to-grasp action. Our understanding of the cortical grasping network continues to depend upon knowledge combined from the different experimental approaches possible in humans and non-human primates. The interrogation of the status of the connections within the grasping network is throwing new light on its operations, and is especially suited for determining the temporal evolution of activity within the network. Indeed we should conclude by emphasising that this network operates on a very fast timescale. The evidence is that visual information about an object can be incorporated into the selected grasping action in around 100–150 ms [50,51^•^]: objects therefore ‘prime’ likely motor responses without a great deal of pre-processing. This fast timescale is a challenge for fMRI studies, because although grasp-specific or reach-specific changes in BOLD will be detected, it is important to know that these changes actually reflect fast processing within the visuomotor circuits. Finally, there is also evidence that a different network, involving areas of the ventral stream, operates when memory-based information is used to guide the grasp [52,53].

## References and recommended reading

Papers of particular interest, published within the annual period of review, have been highlighted as:

• of special interest
•• of outstanding interest
